# Clinical Efficacy of Image-Guided Radiation Therapy for Cervical Cancer and Its Impact on Patients' Serum Tumor Markers and KPS Scores

**DOI:** 10.1155/2022/8536554

**Published:** 2022-07-13

**Authors:** Xin You, Feng-Yan Hou

**Affiliations:** Department of Oncology, Affiliated Hospital of Heibei Engineering University, Handan, China

## Abstract

**Objective:**

To assess the clinical efficacy of image-guided radiation therapy (IGRT) for cervical cancer and its impact on patients' serum tumor markers and Karnofsky Performance Status (KPS) scores.

**Methods:**

Between August 2018 and July 2020, 94 patients with cervical cancer diagnosed and treated in our hospital were recruited and assigned via the random number table method to receive either IGRT (study group) or conventional radiotherapy (control group), with 47 cases to each group. The primary endpoint was clinical efficacy, and secondary endpoints included serum tumor markers levels and KPS scores.

**Results:**

IGRT was associated with a significantly higher efficacy (97.87%) versus convention radiotherapy (74.46%) (*P* < 0.05). IGRT resulted in significantly lower levels of squamous cell carcinoma antigen (SCC-Ag), carcinoembryonic antigen (CEA), carbohydrate antigen 50 (CA50), and carbohydrate antigen 724 (CA724) versus conventional radiotherapy (*P* < 0.05). The eligible patients after IGRT showed significantly higher KPS scores versus conventional radiotherapy (*P* < 0.05).

**Conclusion:**

IGRT enhances the survival of patients with cervical cancer, lowers their serum tumor marker levels, and elevates the KPS scores. Further clinical trials are, however, required prior to clinical promotion.

## 1. Introduction

Cervical cancer [1] is a gynecologic malignancy that originates in the cervical region of the uterus with the second highest incidence and mortality worldwide [2]. The disease mostly develops from the persistent infection with high-risk HPV [3], with insidious early symptoms, and disease progression will lead to symptoms such as fluid discharge and vaginal bleeding. Statistics show a decrease in the average age of onset of cervical cancer [4]. According to the WHO report, 84% of global cases are found in less economically developed countries [5]. Early stage cervical cancer is usually treated with surgery or radiotherapy, while patients with locally advanced cervical cancer are inoperable due to their susceptibility to postoperative recurrence and metastasis, resulting in poor efficacy and survival. Thus, radiotherapy is the treatment of choice for advanced cervical cancer [6]. Radiotherapy [7] is used to kill or inhibit the growth of cancer cells by high-energy rays, and conventional radiotherapy for cervical cancer mostly requires the combination of external beam radiation therapy and internal radiotherapy. External beam radiation therapy uses high-energy rays generated by a radiation machine to kill tumor cells from outside of the body [8, 9], and internal radiotherapy, also called brachytherapy, delivers a high dose of radiation directly to tumor through a specially designed container [10]. Conventional radiotherapy usually causes high doses of radiation to normal tissues such as the small intestine, bladder, and rectum, resulting in a high incidence of side effects and radiation damage (especially in patients with a thin body type or a history of smoking or pelvic infections) [11, 12]. Image-guided radiotherapy (IGRT) uses cone-beam CT (CBCT) to obtain image information in the three-dimensional direction, which is calibrated to acquire the positional error, and reduces the target area error by timely calibration of the six-dimensional bed, which allows precise radiation at tumor [13, 14]. IGRT is a new radiotherapy technique based on 3D conformal radiotherapy with the incorporation of the concept of time, which can adjust the whole external irradiation process by image guidance in real-time to reduce the target area error, and is a significant progress in improving the accuracy of individualized radiotherapy. The present study aims to assess the clinical efficacy of IGRT for cervical cancer and its impact on patients' serum tumor markers and KPS scores and to provide a clinical basis for treatment.

## 2. Materials and Methods

### 2.1. Baseline Data

Between August 2018 and July 2020, 94 patients with cervical cancer diagnosed and treated in our hospital were recruited and assigned via the random number table method to receive either IGRT (study group) or conventional radiotherapy (control group), with 47 cases to each group. The clinical baseline characteristics of the study group (aged 38–70 years, mean age of (45.17 ± 6.72) years, 35 cases of squamous cell carcinoma, 12 cases of adenocarcinoma, 17 cases of International Federation of Gynecology and Obstetrics (FIGO) stage IIb, 14 cases of IIIa, 15 cases of stage IIIb, and 1 case of stage IVa) were comparable with those of the control group (aged 39–68 years, mean age with 47 cases to each group (45.23 ± 6.15) years, 33 cases of squamous cell carcinoma, 14 cases of adenocarcinoma, 18 cases of FIGO stage IIb, 12 cases of IIIa, 15 cases of IIIb, and 2 cases of IVa) (*P* > 0.05) (Table 1). The research was approved by the Ethics Committee of the Affiliated Hospital of Heibei Engineering University.

### 2.2. Inclusion and Exclusion Criteria

#### 2.2.1. Inclusion Criteria

Patients who were diagnosed with cervical cancer confirmed by pathological histology, without secondary primary tumor, and who provided written informed consent were included.

#### 2.2.2. Exclusion Criteria

Patients with serious cardiac, hepatic, renal, and other medical diseases; with an expected survival of >3 months; and without relevant treatment contraindications were excluded.

### 2.3. Treatment Methods

The control group received conventional radiotherapy. The 15 MV X-ray simulator was selected for positioning, and 0° and 180° anterior-posterior pelvic counter irradiation fields were used to create digitally reconstructed radiographs (DRR) on the planning system, and a full pelvic irradiation field (ranging from the upper border between the 4th and 5th lumbar vertebrae, the lower border at the inferior border of the pubic symphysis, and the two lateral borders at the level of the anterior superior iliac spine) was designed according to the bony landmarks, with conventional fractionation of 1.8–2.0 Gy per session, 5 times per week, a whole pelvic dose of (30–45) Gy/(3–4.5) W.

The study group received IGRT. CT simulator isocentric point calibration and Varian RapidArc machine treatment placement were performed prior to radiotherapy. The Elekta Synergy IGRT system was used to analyze the interfraction and intrafraction positioning errors by using the kilovoltage CBCT on board, and the CBCT 3D reconstructed volume images obtained after the first six (once a day) and the last four (once a week) positions of the patient were matched with the positioning CT images to automatically display the deviation of the patient's actual target center position from the treatment scheduled target center position in the 3D direction, followed by the online alignment with the preradiotherapy positioning image. The radiotherapy plan based on preradiotherapy localization CT was fused with the images at each CBCT treatment and the dose distribution of the target area and organs at risk such as the bladder and rectum. The sigmoid colon was compared offline between the two plans to further clarify the location and dose deviation and to determine any need for treatment replanning to improve radiotherapy accuracy. The IGRT P-CTV dose was 45 Gy/25f.

### 2.4. Clinical Endpoints

Efficacy: the efficacy was evaluated by referring to the Response Evaluation Criteria in Solid Tumors (RECIST) [15], including complete response (CR), partial response (PR), stable disease (SD), and progressive disease (PD). CR: all tumor lesions disappeared, and no new lesions appeared in 4 weeks. PR: the total longest diameter of the lesions at baseline was reduced by ≥30% for 4 weeks. SD: the total longest diameter of the lesions at baseline was reduced by less than 30%. PD: the total longest diameter of lesions increased by ≥20%, or one or more new lesions appeared. Efficacy = (CR cases + PR cases)/total cases × 100%.

Serum tumor markers: 2 ml of fasting venous blood was collected from patients before and after treatment, respectively, and centrifuged at 3000 r/min for 10 min after resting for 1 h. The supernatant was collected for the determination of serum tumor markers, including squamous cell carcinoma antigen (SCC-Ag, SEB372Hu02), carcinoembryonic antigen (CEA, K4805-100), carbohydrate antigen 50 (CA50, CSB-EQ027409HU), and carbohydrate antigen 724 (CA724, 0-003371), using an enzyme-linked immunosorbent kit (Beijing Kangtai Biotechnology Co. Ltd.)

Karnofsky Performance Status (KPS) score [16]: the KPS score was used to evaluate the performance of patients before and after treatment. The score was divided into 10 levels according to the patients' condition and degree of self-care, with a score of 10 points for each level and a total score of 100 points. A score of <60 points indicate poor health status and poor quality of life, and the higher the score, the better the quality of life of patients.

### 2.5. Statistical Analysis

SPSS 22.0 was used for data analyses. The measurement data are expressed as (mean ± SD) and analyzed using the *t*-test. The count data are expressed as the number of cases (rate) and analyzed using the chi-square test. Differences were considered statistically significant at *P* < 0.05.

## 3. Results

### 3.1. Clinical Efficacy

IGRT was associated with a significantly higher efficacy (97.87%), including 28 (59.57%) cases of CR, 18 (38.29%) cases of PR, 1 (2.13%) case of SD, and 0 cases of PD, versus convention radiotherapy (74.46%, 19 (40.42%) cases of CR, 16 (34.04%) cases of PR, 9 (19.15%) cases of SD, and 3 (6.38%) cases of PD) (*P* < 0.05) (Table 2).

### 3.2. Serum Tumor Markers

Before treatment, the two groups showed similar levels of SCC-Ag, CEA, CA50, and CA724 (*P* > 0.05). IGRT resulted in significantly lower levels of SCC-Ag, CEA, CA50, and CA724 (1.06 ± 0.13, 1.13 ± 0.24, 15.45 ± 2.51, and 1.74 ± 0.31) versus conventional radiotherapy (2.44 ± 0.24, 3.48 ± 0.27, 24.65 ± 2.96, and 7.95 ± 1.23) (*P* < 0.05).

### 3.3. KPS Scores

There were no significant differences in the pretherapy KPS scores between the two groups (*P* > 0.05). The eligible patients after IGRT showed significantly higher KPS scores (85.17 ± 9.24) versus conventional radiotherapy (74.46 ± 8.87) (*P* < 0.05) (Table 4).

## 4. Discussion

It has been reported that 70% of patients with cervical cancer are caused by HPV type 16 and HPV type 18 [15]. The 2015 NCCN guidelines [16] state that surgery is mostly used for the radical treatment of patients with early stage cervical cancer, while radiotherapy is the mainstay of treatment for advanced cervical cancer. However, conventional radiotherapy usually predisposes to multiple complications. IGRT [17] is a four-dimensional radiotherapy technique that incorporates the movement of anatomical tissues during treatment and the displacement error between fractional treatments and uses various advanced imaging devices to monitor tumors and normal organs in real-time before and during treatment to achieve precise radiotherapy [18, 19]. The results of Wang et al. showed that IGRT significantly improved the efficacy of cancer treatment [13]. The clinical objective of cervical cancer treatment is to achieve tumor local control and reduce associated complications. Franzone et al. [20] found that IGRT showed excellent efficacy and a low incidence of long-term adverse effects in prostate cancer patients.

In the present study, IGRT was associated with a significantly higher efficacy versus convention radiotherapy. The reason may be that IGRT can regulate the intensity of irradiation dose to the tumor target area and adjacent sensitive organs, which can accelerate the dose gradient decrease, increase the local dose to the tumor, form a concave target area dose distribution to enhance tumor control, effectively improve the accuracy of radiation therapy, and minimize collateral damage, thereby preventing complications and enhancing the treatment efficacy. Some studies also reported that IGRT can substantially improve local progression-free survival and five-year overall survival in cancer patients. SCC-Ag is the preferred serological tumor marker for cervical squamous carcinoma, and CEA, CA50, and CA724 are metabolites of tumor cells or tumor-host cells, which possess a certain clinical application value for early diagnosis and prognosis assessment of malignant tumors. Relevant studies have shown abnormally high expression of SCC-Ag, CEA, CA50, and CA724 in serum of patients with cervical cancer. The results of the present study showed that IGRT resulted in significantly lower levels of SCC-Ag, CEA, CA50, and CA724 versus conventional radiotherapy, which may be attributed to the fact that IGRT performs calibration of position and dose distribution to reduce positional errors, increases the irradiation dose to the tumor target area, and reduces the exposure of normal tissue, which kills cancer cells while effectively inhibiting DNA repair, thereby controlling cancer cell proliferation and suppressing the metastasis of tumor cells and lesion infiltration. Moreover, patients receiving IGRT in this study showed significantly higher KPS scores versus conventional radiotherapy, indicating a better prognosis of patients and quality of life, confirming a high safety profile of IGRT. Due to the small sample size and the absence of long-term follow-up, a long-term follow-up will be conducted in future studies to observe the long-term outcome of IGRT treatment and the survival of patients to provide a solid and effective reference for the clinical treatment of cervical cancer.

IGRT is an emerging four-dimensional radiotherapy technique in recent years, featuring the advantages of intensity-modulated radiotherapy technology and more emphasis on the consideration of the movement and displacement errors of anatomical tissues during the treatment process. Clinical practice has confirmed that with the support of imaging equipment for real-time monitoring of tumors and normal tissues, IGRT provides the maximum dose to the target area and the minimum dose to the surrounding normal tissues, which meets the basic requirements of precise radiotherapy [1]. It has been found that although the dose distribution within a single radiation field is uneven under IGRT, the target localization and irradiation accuracy outperform general radiotherapy, with a more balanced dose distribution throughout the target volume, which significantly improves the radiotherapy effect and reduces radiation damage [5].

## 5. Conclusion

IGRT enhances the survival of patients with cervical cancer, lowers their serum tumor marker levels, and elevates the KPS scores. Further clinical trials are, however, required prior to clinical promotion.

## Figures and Tables

**Table 1:** Comparison of baseline data ($\bar{x}\pm s$).

Groups	*n*	Age	Pathological type		FIGO stage			
			Squamous cell carcinoma	Adenocarcinoma	IIb	IIIa	IIIb	IVa
Study group	47	38–70 (45.17 ± 6.72)	35	12	17	14	15	1
Control group	47	39–68 (45.23 ± 6.15)	33	14	18	12	15	2
*t* value	—	0.045						
*P* value	—	0.964						

**Table 2:** Comparison of clinical efficacy (%).

Groups	*n*	CR	PR	SD	PD	Total efficacy
Study group	47	28 (59.57)	18 (38.29)	1 (2.13)	0 (0.00)	46 (97.87)
Control group	47	19 (40.42)	16 (34.04)	9 (19.15)	3 (6.38)	35 (74.46)
*x* ^2^	—	10.802				
*P* value	—	0.001				

CR, complete response; PR, partial response; SD, stable disease; PD, progressive disease.

**Table 3:** Comparison of serum tumor markers ($\bar{x}\pm s$).

Groups	Time	Control group (*n* = 47)	Study group (*n* = 47)	*t* value	*P* value
SCC-Ag (ng/ml)	Before treatment	6.53 ± 0.48	6.54 ± 0.39	0.111	0.912
	After treatment	1.06 ± 0.13	2.44 ± 0.24	34.662	<0.001

CEA (*μ*g/L)	Before treatment	8.69 ± 1.08	8.74 ± 1.05	0.228	0.82
	After treatment	1.13 ± 0.24	3.48 ± 0.27	44.598	<0.001

CA50 (U/ml)	Before treatment	45.52 ± 4.21	45.39 ± 4.84	0.139	0.89
	After treatment	15.45 ± 2.51	24.65 ± 2.96	16.252	<0.001

CA724 (U/ml)	Before treatment	28.14 ± 3.79	28.51 ± 3.08	0.519	0.605
	After treatment	1.74 ± 0.31	7.95 ± 1.23	33.563	<0.001

SCC-Ag, squamous cell carcinoma antigen; CEA, carcinoembryonic antigen; CA50, carbohydrate antigen 50; CA724, carbohydrate antigen 724.

**Table 4:** Comparison of KPS scores ($\bar{x}\pm s$).

Groups	*n*	Before treatment	After treatment
Study group	47	70.12 ± 6.23	85.17 ± 9.24
Control group	47	70.08 ± 6.85	74.46 ± 8.87
*t* value	—	0.030	5.733
*P* value	—	0.976	<0.001

## Data Availability

The datasets used during the present study are available from the corresponding author upon request.
